# Associations among PM_2.5_, corticotropin releasing hormone, estriol, and progesterone in pregnant persons in Puerto Rico

**DOI:** 10.1088/2515-7620/adc0f1

**Published:** 2025-03-26

**Authors:** Trenton Honda, Trenton Henry, Christina A Porucznik, Laura Corlin, Kipruto Kirwa, Akram Alshawabkeh, José F Cordero, Carmen M Velez Vega, Zaira Y Rosario Pabon, John D Meeker, Helen Suh

**Affiliations:** 1 School of Clinical and Rehabilitation Sciences, Northeastern University, Boston, MA, United States of America; 2 Spencer Fox Eccles School of Medicine, University of Utah, Salt Lake City, UT, United States of America; 3 Department of Public Health and Community Medicine, Tufts University School of Medicine, Boston, MA, United States of America; 4 Department of Civil and Environmental Engineering, Tufts University School of Engineering, Medford, MA, United States of America; 5 Department of Environmental Health, Boston University School of Public Health, Boston, MA, United States of America; 6 Department of Civil & Environmental Engineering, Northeastern University, Boston, MA 02115, United States of America; 7 Department of Epidemiology, University of Georgia College of Public Health, Athens, GA, United States of America; 8 University of Puerto Rico Medical Sciences Campus, University of Puerto Rico Graduate School of Public Health, San Juan, PR, United States of America; 9 Department of Social Sciences, Graduate School of Public Health, University of Puerto Rico Medical Sciences Campus, San Juan, PR, United States of America; 10 Department of Environmental Health Sciences, University of Michigan School of Public Health, Ann Arbor, MI, United States of America

**Keywords:** particulate air pollution, PM_2.5_, pregnancy, hormones, progesterone

## Abstract

*Background.* Exposure to PM_2.5_ is associated with adverse birth outcomes and early development. Pregnancy is typically characterized by the production of several important hormones that impact aspects of maternal and fetal physiology, including progesterone, estriol, and corticotropin releasing hormone (CRH). No previous studies have examined PM associations in pregnant persons for CRH and estriol. *Methods.* We used linear mixed effects models to investigate associations between PM_2.5_ and pregnancy hormones in 1,041 pregnant persons ages 18–41 living in Puerto Rico between 2011 and 2020. Individual 3–, 7–, and 30-day moving average exposures were assigned from EPA data sources. Hormone levels were analyzed in blood collected at study visits at 16–20 and 20–24 weeks of gestation. Models were adjusted for demographics, socioeconomic status, and health behaviors.*Results.* Mean participant exposures for 3−, 7−, and 30-day PM_2.5_ were 8.0 ± 5.9, 8.2 ± 5.3, and 8.1 ± 4.4 μg m^−3^. In base models, increased PM_2.5_ exposure was associated with lower levels of progesterone, CRH, and estriol. In adjusted models, 10 μg m^−3^ increase in PM_2.5_ was associated with 11.2% (95% CI: 17.6, 4.3; p = 0.003) and 14.9% (95% CI: 23.4, 5.4; p = 0.004) lower CRH for 7-day and 30-day exposures. In cross-sectional models, the inverse CRH association was driven by the 20–24 week gestation period with a 12.4% reduction (95% CI: 21.8, 1.9; p = 0.022) for 7-day and 17.5% reduction (95% CI: 29.7, 3.0; p = 0.020) for 30-day exposure. Other investigated associations were null.*Conclusions.* In pregnant persons in Puerto Rico, we observed that elevated PM_2.5_ exposures were significantly associated with decrements in CRH, but not in other pregnancy-associated hormones. CRH may be an important pathway through which prenatal PM_2.5_ impacts normal pregnancy.

## 1. Introduction

The U.S. spends more on pregnancy and birth-related healthcare than any other developed country [1]; however, rates of adverse birth outcomes in the US (e.g., preterm births, low birthweight, infant and maternal mortality) are among the developed world’s highest [2, 3]. Important contributing factors to these high rates include a history of systemic racism and exclusion of some groups (e.g., non-Hispanic Black and Hispanic white maternal mortality rates exceed non-Hispanic white rates), socioeconomically disadvantaged geography (e.g., low birthweight correlates with ethnoracial disparity), and low socioeconomic status (e.g., preterm birth rates are higher among low-income households) [4–6]. Such social determinants have also been shown to be linked to increased exposure to harmful environmental toxicants like air pollution [7].

Air pollutants, such as PM_2.5_, have previously been linked to several adverse birth outcomes, including fetal developmental pathologies, low birthweight, preterm birth, and infant and neonatal mortality—outcomes that are more common amongst underrepresented and historically-excluded communities [8–14]. For example, Woo *et al* found that both residential segregation and racial/ethnic disparities are associated with significant increases in exposure to ambient particulate pollution (and other toxicants), particularly among Black and Latino populations [15]. Zip code-level analysis further support this finding, with higher exposure levels observed in Black, Asian and Hispanic or Latino as compared to non-Hispanic white population areas [16]. The intersection of higher pollution exposure and social determinants of health, which increases the risk of adverse birth outcomes, emphasizes the need to study underrepresented communities that bear an undue burden of poor health.

The US archipelago territory of Puerto Rico is such a community, with a primarily Hispanic population (exceeding 98%), a poverty rate of 41.7%, and a median household income of $21,967 (compared to the mainland USA’s $69,021 based on 2022 estimates ) [17, 18]. Chronic illness degrades health-related quality of life for Puerto Ricans and is likely linked to systemic racism and discrimination [19–21]. Adverse maternal and fetal health outcomes are also quite high: low birthweight and preterm birth rates exceed those observed on the US mainland [22], maternal mortality is higher than in other Hispanic populations [23], and infant mortality exceeds non-Hispanic white levels [24]. While air pollution levels often meet EPA standards on the island, the impact of air pollutants on health in Puerto Rico remains poorly studied despite known causes for concern for excess environmental pollution, such as industrial production, coal-burning power plants, and volcanic dust carried by trade winds. All these variables may contribute to the particulate air pollution in Puerto Rico having greater toxicity [25, 26].

The mechanisms through which PM_2.5_ may impact fetal growth and development are not completely understood. Animal models suggest that specific gestational timeframes are important determinants of outcomes [27], perhaps due to maternal systemic inflammation resulting from alveolar macrophage degranulation and/or particles translocating directly into the maternal circulation and impacting placental health [28–30]. Indeed, particulate matter components (such as black carbon) have been observed in the placenta, supporting the role of particle translocation as a mechanism of altered placental health [31]. Placental health is imperative not only due to its direct role in providing nutrients in support of fetal development, but also its role in the production of several pregnancy-regulating hormones, including progesterone, estriol, and corticotropin releasing hormone (CRH).

Progesterone is a critical hormone in the maintenance of pregnancy, fetal development, and parturition [32, 33]. Low progesterone levels during pregnancy have been linked to increased risk of spontaneous abortion and low birthweight, and perturbations in pregnancy duration [34–36]. Although the role of CRH in pregnancy is well understood, [37–40] especially for parturition, pollution effects on CRH and maternal and fetal health are less well defined. Placental CRH has been linked to fetal glucose metabolism and neurodevelopment, post-natal adiposity, and pre/post-term labor [41–46]. Importantly, prior literature also suggests that CRH helps regulate progesterone production in placental cells—with CRH rising throughout pregnancy and quickly tapering after birth. This may implicate CRH production as a pathway by which PM_2.5_ can affect several pregnancy outcomes [32, 47]. Estriol (E3) accounts for 90% of the estrogens that circulate during pregnancy [48–50], and stimulates uterine growth, placental development, and preparation for parturition. Although animal studies suggest that PM_2.5_ exposure is associated with dysregulation of mammalian estrogen receptors, and human studies indicate pollution-related decreases in the related hormone estradiol at other life stages, PM’s effect on estriol during human pregnancy is unclear [51, 52].

Whether maternal exposure to PM_2.5_ impacts the production of these key pregnancy-regulating hormones, and what biological pathways may best explain this impact, remain largely unexplored, particularly in populations impacted by significant socioeconomic disadvantage. Thus to address these gaps in the literature, we investigated whether maternal PM_2.5_ exposure is associated with perturbations in maternal progesterone, CRH, and estriol levels in a cohort of pregnant persons in Puerto Rico.

## 2. Methods

### 2.1. Study design and population

The ongoing Puerto Rico Testsite for Exploring Contamination Threats (PROTECT) prospective cohort study seeks to investigate environmental impacts on birth outcomes among Puerto Rico’s pregnant persons residing in its north karst region. We identified PROTECT participants recruited between years 2011 and 2020 who contributed blood samples with measures for progesterone, CRH, and estriol. The study’s design has been described previously in detail [53, 54] and involved up to three prenatal clinic visits (visit 1 at 16 to 20 weeks, visit 2 at 20 to 24 weeks, and visit 3 at 24 to 28 weeks). Visits 1 and 3 collected blood (which provided biosamples of pregnancy hormones) and urine spot samples, whereas visit 2 collected only spot urine (and therefore did not contain samples of pregnancy hormones). Data were also collected on participants’ demographic characteristics, clinical history, behavioral information, and pregnancy status. Visit timing aligns with the primary goal of PROTECT, which was to investigate the possible effects of environmental exposures on shortened gestational length leading to preterm birth (defined as birth before 37 weeks). The range of preterm births is from 24 to 37 weeks. Therefore, the first visit was scheduled at 16 to 20 weeks gestation—the early part of the second trimester (14 to 27 weeks). The second visit was scheduled for 20 to 24 weeks, which overlaps the second semester. The third visit covers the end of the second trimester and the beginning of the third trimester (28 to 40 weeks).

### 2.2. Institutional review board

The PROTECT study has IRB approval from the University of Puerto Rico, Northeastern University, University of Michigan, and the University of Georgia. This study was approved by the Northeastern University Institutional Review Board IRB #21-10-18.

### 2.3. Exposures

The exposure of interest was fine particulate matter ≤ 2.5 micrometers in diameter (PM_2.5_), which was gathered across 13 island sensors by the US Environmental Protection Agency (EPA) air quality system (AQS) and retrieved from the AQS data mart [55]. Using 24 h mean samples pre-calculated by the EPA and documented further in ‘Daily Summary Files content’ [56], exposures were constructed for 3−, 7−, and 30-day moving averages preceding each participant’s study visits 1 and 3, and linked to the centroid of the participant’s residential municipality using inverse distance weighting (squared), IDW^2^ [57]. Additionally, 1− and 3-day lags were calculated for the 3-day moving average exposures. PM_2.5_ exposures were scaled to represent differences in outcome per each increase of 10 μg m^−3^. We conducted sensitivity analyses that limited participant distances from monitors and compared the effect of inverse-distance-weighting-cubed interpolation.

### 2.4. Outcomes

Details of serum hormone analysis has been described previously [58, 59]. Serum samples were analyzed at the Central Ligand Assay Satellite Services (CLASS) laboratory in the Department of Epidemiology at the University of Michigan School of Public Health. Progesterone was measured using a chemiluminescence immunoassay, while estriol and corticotropin releasing hormone were measured using an enzyme immunoassay. Some hormone concentrations were not available for all participants due to sample volume limitations. Concentrations of progesterone, CRH, and estriol were log-transformed due to their skewed distributions to accommodate linear modeling. For both outcomes, lab analyses that fell below the limit of detection (LOD) were assigned the value LOD/√2 [60] (LOD for CRH was 4.9 ng ml^−1^; LOD for progesterone was 0.1 ng ml^−1^; LOD for estriol was 0.22 ng ml^−1^).

### 2.5. Covariates

Covariates and categorical cut points were selected *a priori* based on previous analysis of this cohort [12]. They included demographic information [maternal age (continuous], indicators of socioeconomic status [employment status (categorical), household income (categorical), education (categorical)], medical history and health behaviors [parity (categorical), exercise (categorical), BMI (categorical), alcohol consumption (categorical), smoking status (categorical)], marital status (categorical), municipality (categorical), visit month (categorical), gestational age (continuous), and batch (processing date) of lab hormone analysis (categorical).

### 2.6. Statistical analysis

#### 2.6.1. Models

Linear mixed effects models with random intercepts at the level of the participants were used to determine associations between ambient PM_2.5_ and natural-log-transformed progesterone, CRH, and estriol [61]. Fully adjusted models included age, education, marital status, parity, history of adverse pregnancy outcome, employment status, household income, race, exercise, BMI, alcohol consumption, smoking status, municipality, visit month, gestational age, and batch processing date of lab hormone analysis. Beta coefficients were exponentiated to provide percent difference in effect per 10 μg m^−3^ exposure.

In sensitivity analyses, we examined cross-sectional associations at each clinic visit and performed complete-case (non-imputed) analyses. Additionally, we assessed collinearity of covariates and conducted sensitivity analyses of models using covariate subsets.

#### 2.6.2. Missing data and imputation

We used multiple imputation with chained equations to create 30 imputed data sets with non-missing covariates [62]. PM_2.5_ and analyte values were not imputed. Regression was performed on all 30 data sets, and effect estimates were pooled to obtain final effect estimates [63]. Estimates were exponentiated and converted to percentages. Missing data levels prior to imputation are shown in Supplemental table 1.

#### 2.6.3. Software

All analyses were performed using R Statistical Software version 4.2.2 with packages MICE, lme4, merTools, and mediation [64].

## 3. Results

Table 1 shows the demographic characteristics of the 1,041 participants. Their average age was 26.9 ± 5.5 years. More than 65% had some postsecondary education, but over 60% had household incomes <$30,000 per year. During the study period of 2011–2020, the mean participant exposures for 3-, 7-, and 30-day PM_2.5_ were 8.0 ± 5.9, 8.2 ± 5.3, and 8.1 ± 4.4 μg m^−3^, respectively. Average participant progesterone levels for visits 1, 3, and mean pregnancy were 44.8, 86.3, and 62.0 ng ml^−1^, respectively; mean participant CRH levels for visits 1, 3, and mean pregnancy were 53.5, 53.8, and 53.6 ng ml^−1^, respectively; for E3, means at the same timeframes were 18.6, 42.6, and 28.6 ng ml^−1^.

**Table 1.** Demographic and pregnancy characteristics of PROTECT participants.

Characteristic	N = 1,041^1^
Mother’s age (years)	26.9 ± 5.5
Employed at first study visit	
Yes	644 (62.2%)
No	390 (37.7%)
Refuse to answer	1 (0.1%)
Mother’s Race	
Hispanic White	523 (53.8%)
Mestiza	404 (41.6%)
Black	29 (3.0%)
Multi-racial	16 (1.6%)
Household income per year, US dollars	
< $30,000	587 (64.0%)
≥ $30,000	330 (36.0%)
Mother’s education	
Less than high school	230 (22.1%)
High school or technical school	128 (12.3%)
College	681 (65.5%)
Number of pregnancies, including this one	
1	451 (43.4%)
2	371 (35.7%)
3 or more	218 (21.0%)
Number of other children, excluding this one	
0	375 (42.2%)
1	391 (44.0%)
2 or more	122 (13.7%)
Exercise habits	
Not exercised for > 30 min day^−1^ in past 3 months	835 (80.4%)
Exercised for > 30 min day^−1^ in past 3 months	202 (19.4%)
Do not know	2 (0.2%)
Maternal pre-pregnancy BMI	
Normal or underweight	432 (44.6%)
Overweight	313 (32.3%)
Obese	224 (23.1%)
Alcohol consumption	
Alcohol consumed before pregnancy	428 (41.3%)
Alcohol consumed during pregnancy	66 (6.4%)
No alcohol use	542 (52.3%)
Any cigarette smoke exposure in the home	
No smoke exposure	857 (87.4%)
Smoke exposure	124 (12.6%)
Marital status at first study visit	
Married	549 (52.9%)
Cohabiting	275 (26.5%)
Single or divorced	211 (20.3%)
Other	2 (0.2%)
^1^ n (%); Mean ± SD; excludes missing values	

**Figure 1.**
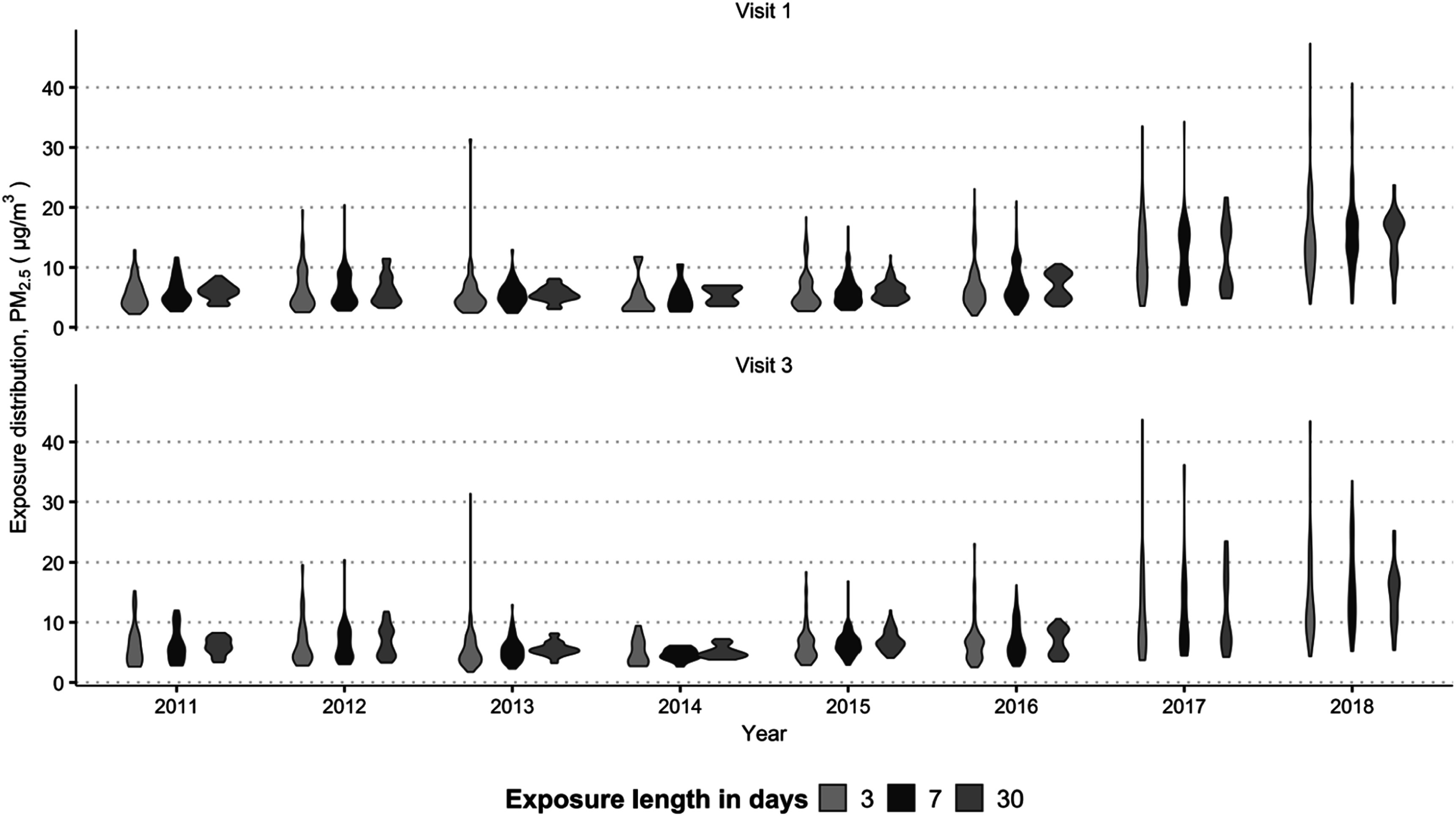
Participant PM_2.5_ exposures during study years, by visit and exposure length.

Figure 1 shows the distribution of participant PM_2.5_ exposure over the study duration.

Sensitivity analyses using monitor distance cutoffs of < 51, < 26, and < 11 km (Supplemental tables 3–5). While associations were not importantly changed for <51 and <26 km, restricting to smaller distances dramatically decreased power owing to the smaller sample size. Sensitivity analysis of inverse distance weighting cubed (IDW^3^) interpolation (Supplemental table 6), which has been successfully employed in other geospatial and epidemiological work [65, 66], shows an overall trend of away from the null.

**Figure 2.**
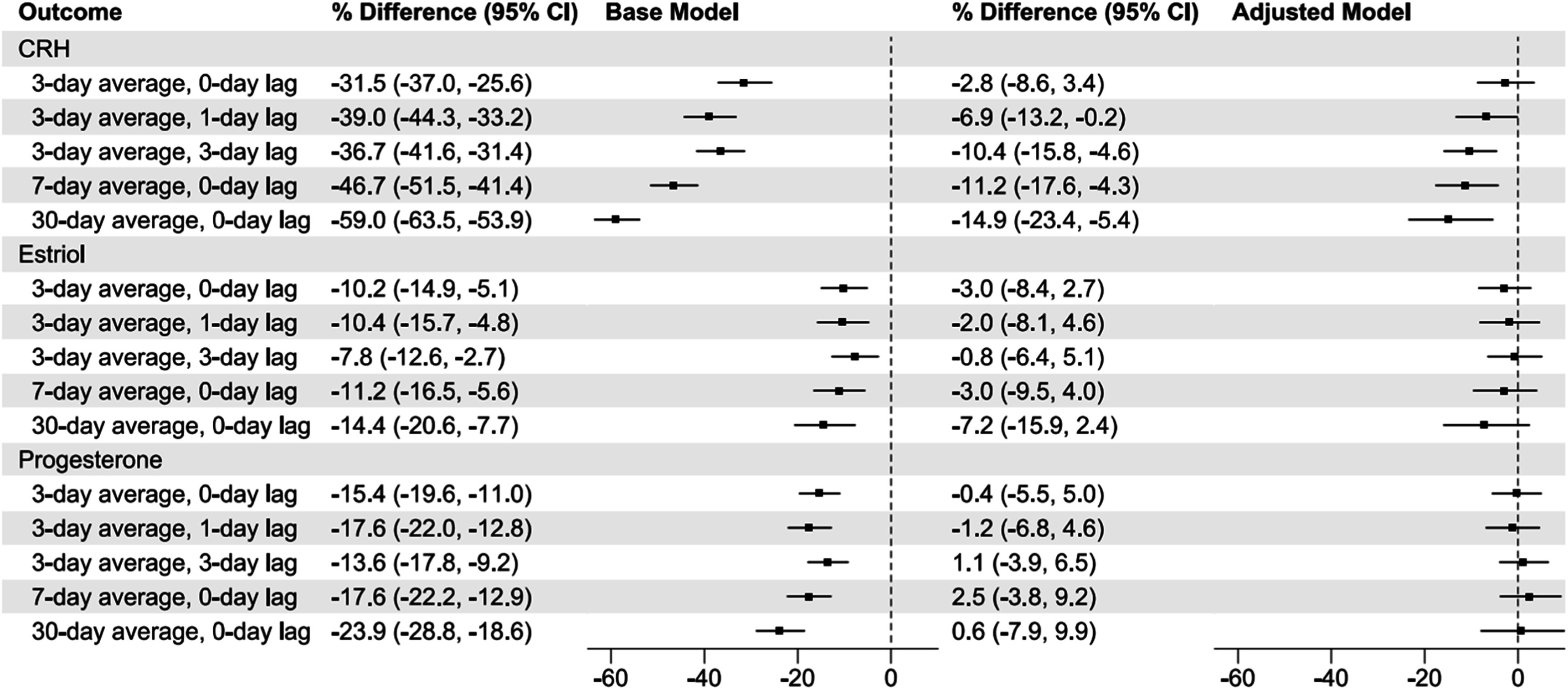
Associations between 10 μg m^−3^ increase in ambient PM_2.5_ and maternal hormone levels: Mixed effects models over all visits (both visits 1 and 3 as repeat measures). Model adjusted for education, age, marital status, parity, prior negative pregnancy outcomes, job status, income, race, exercise, BMI, alcohol use, smoking exposure, municipality of residence seasonality (month), gestational age, and batch (date) of hormone lab analysis.

Figure 2 shows associations between PM_2.5_ and pregnancy hormones for 3−, 7−, and 30-day moving averages with additional lags for sub-week exposures. In unadjusted mixed-effects models (using visits 1 and 3 as repeat measures), we observed inverse associations for all moving average exposures and lags for CRH, estriol, and progesterone. In fully adjusted models, significant associations were observed in CRH levels for 3-day moving average exposures with 1- (% Difference: −6.9, 95% CI: −13.2, −0.2) and 3- (% Difference: −10.4, 95% CI: −15.8, −4.6) day lags. Stronger associations were observed for longer term moving averages. For example, the largest magnitude association was observed for 30-day moving average exposures, where a 10 μg m^−3^ increase in ambient PM_2.5_ was associated with a 14.9% (95% CI: 23.4, 5.4) lower CRH level.

**Figure 3.**
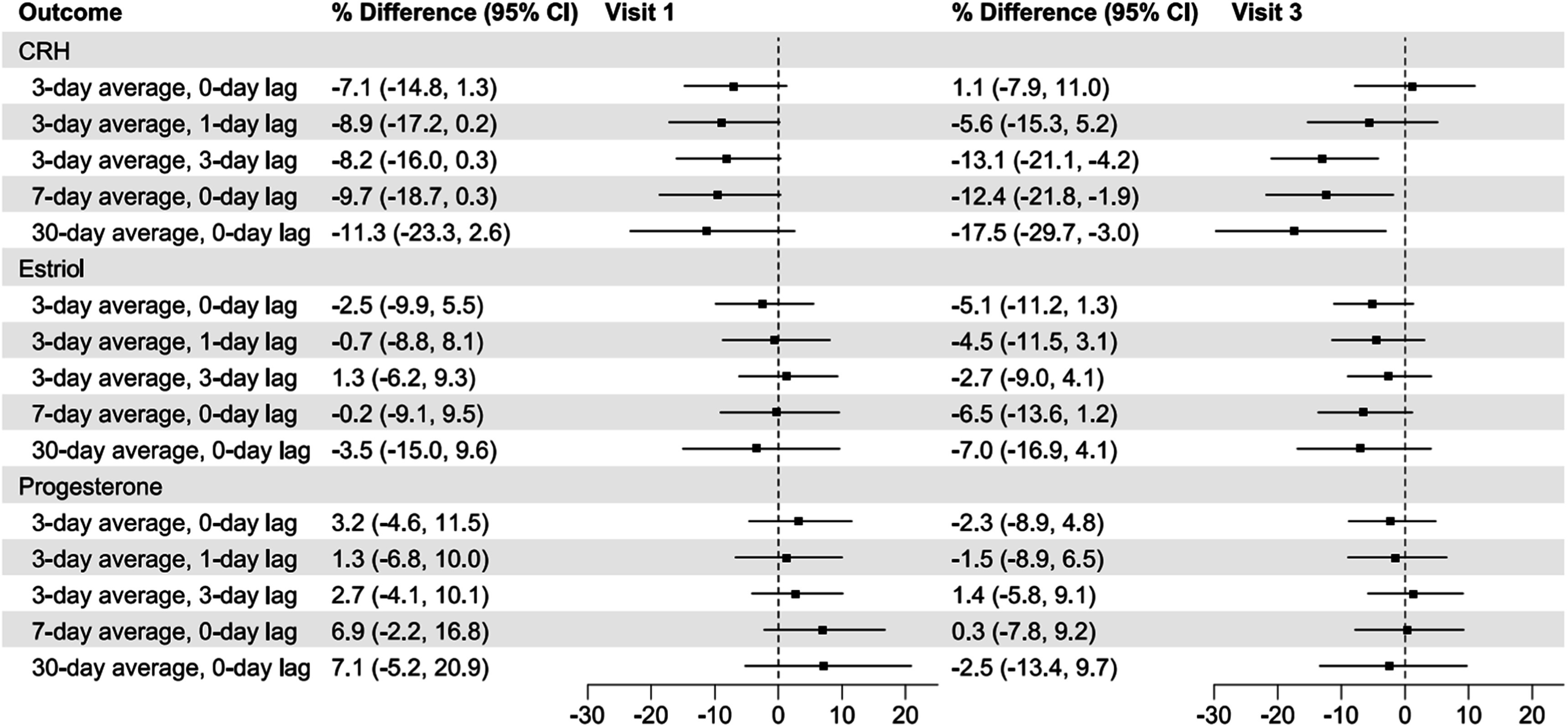
Cross-sectional associations between 10 μg m^−3^ increase in ambient PM_2.5_ and maternal hormone levels for visits 1 and 3. Model adjusted for education, age, marital status, parity, prior negative pregnancy outcomes, job status, income, race, exercise, BMI, alcohol use, smoking exposure, municipality of residence seasonality (month), gestational age, and batch (date) of hormone lab analysis.

Figure 3 shows fully adjusted cross-sectional associations between PM_2.5_ and pregnancy hormones for visits 1 and 3. Notably, significant changes to CRH levels are attributable to exposures preceding visit 3 (later pregnancy) rather than visit 1 (early pregnancy).

No significant effect modification of the associations between PM and pregnancy related hormones was observed by BMI (underweight/normal, overweight, obese) or maternal age (data not shown). Sensitivity analyses of complete cases (Supplementary table 2) and excluding BMI from models did not meaningfully change our results (Supplemental table 3). Although covariates were selected for comparability with previously published research on this cohort, and variance inflation was shown to be < 3 for all variables in fully adjusted models using the mean of the imputed measures (Supplemental figure 1). We further conducted a sensitivity analysis that included only statistically significant (*α* = 0.05) coefficients (Supplemental table 8) which did not importantly change our effect estimates. In addition, supplemental table 9 shows the relationship between quartiles of 30-day PM2.5 exposure, concomitant hormone levels, and prevalence of pre-term birth in this cohort.

## 4. Discussion

To our knowledge, this is the first study to identify an association between PM_2.5_ exposure and lowered CRH within a birth cohort. We observed large magnitude and consistent effects for most exposure windows, with the strongest associations observed for the 30-day exposure period in our mixed effects models, which were consistent with our visit 3 (20–24 week gestational age) cross-sectional analysis. For other pregnancy related hormones (i.e., progesterone and estriol) we observed no significant associations.

Prior literature on the impact of prenatal air pollution exposure and pregnancy related hormones is mixed. Several studies have shown PM associations with negative birth outcomes, while few have explored progesterone as a potential pathway with mixed results. A systematic review by Li *et al* identified possible pathways by which air pollution may impact birth outcomes, including: oxidative stress, DNA methylation, alteration of mitochondrial DNA, and disruption of normal endocrine pathways [67]. Accordingly, in an *in vitro* study Wang *et al* [68] found that introducing 1 and 5 mg ml^−1^ of PM_2.5_ to human trophoblast cells over 48 h caused a significant decrease in progesterone production, compared to control cells. Similarly, that study found that industry-derived PM_2.5_ suppressed progesterone production in a trophoblastic placental cell line, while Tao *et al* found that PM exposure diminished maternal serum androgens, but did not affect progesterone levels in a rat model [69]. Interestingly, Naav *et al* found that short term PM_2.5_ exposure to a placental trophoblastic cell line led to accumulation of particulates within the cell cytoplasm and induced markers of cytotoxicity and cellular death, but also found that progesterone was increased in the short (48 h) timeframe after exposure, which is inconsistent with the prior *in vitro* literature, and our epidemiologic study in human subjects [70].

To our knowledge, no prior studies have examined the impact of PM_2.5_–or other air pollutants–on CRH. Importantly, CRH is associated with normal neurodevelopment, and interacts with both the maternal and fetal stress responses to maintain physiologic homeostasis [44]. We found significant and consistent decrements in CRH level associated with ambient PM_2.5_ exposure, which has been previously associated with placental inflammation and ventriculomegaly in premature infants [43, 44, 71]. This is consistent with our prior work in this study cohort, wherein we found PM components to be associated with perturbations in infant non-nutritive suck, an emerging marker of neurological integrity [72]. Conversely, we found no association between air pollution exposure and estriol or progesterone levels. Both estriol and progesterone are placental products with important roles in maintenance of pregnancy and parturition [32, 33, 48–50]. Why we observe associations with CRH, but not these other critical pregnancy related hormones, remains unclear.

Our study has a number of important limitations. First, Puerto Rico suffered several climate-related, island-wide power failures during the study period, which resulted in some missing exposure data. However, because missing data occurred for all participants during these episodes, we do not expect differential misclassification bias. Second, as modeled exposure estimates are unavailable for Puerto Rico, exposure assignment was limited to the municipality participants lived in which may introduce exposure misclassification. Third, ambient PM exposure does not account for daily movement or time spent indoors which may contribute further to exposure error.

These limitations are counterbalanced by several study strengths. Ours is the first study to examine the impact of ambient PM on three critical pregnancy-related hormones in Puerto Rico. Our use of an established, longitudinal birth cohort provides us access to high-quality medical history, covariate, and biomarker data. Finally, this study helps to address knowledge gaps in an understudied and disadvantaged population whose burden of disease is quite high.

## 5. Conclusions

We observed that elevated PM_2.5_ exposures were significantly associated with decrements in CRH in pregnant persons in Puerto Rico. Our findings suggest that PM_2.5_ exposure may result in perturbations in endocrine physiology of pregnancy which is a potential mechanism by which PM exerts deleterious effects on fetal and neonatal birth outcomes.

## Data Availability

The data cannot be made publicly available upon publication because they contain sensitive personal information. The data that support the findings of this study are available upon reasonable request from the authors.

## References

[1] (OECD) (2021). O.f.E.C.-o.a.D. Infant mortality rates. https://data.oecd.org/healthstat/infant-mortality-rates.htm.

[2] National Academies of Sciences, E., and Medicine (2020). Birth Settings in America: Outcomes, Quality, Access, and Choice.

[3] (WHO), W.H.O (2023). Maternal, newborn, child and adolescent health and ageing - data portal. https://platform.who.int/data/maternal-newborn-child-adolescent-ageing/indicator-explorer-new/.

[4] Hoyert D (2023). Maternal mortality rates in the United States.

[5] Douds K W, Raker E J (2021). The geography of ethnoracial low birth weight inequalities in the United States. SSM Popul Health.

[6] Brumberg H L, Shah S I (2015). Born early and born poor: an eco-bio-developmental model for poverty and preterm birth. J. Neonatal. Perinatal. Med..

[7] U.S. Department of Health and Human Services, H (2023). Healthy People 2030.

[8] Arroyo V (2016). Short term effect of air pollution, noise and heat waves on preterm births in Madrid (Spain). Environ. Res..

[9] Ashrap P (2020). Maternal blood metal and metalloid concentrations in association with birth outcomes in Northern Puerto Rico. Environ. Int..

[10] Goyal N, Karra M, Canning D (2019). Early-life exposure to ambient fine particulate air pollution and infant mortality: pooled evidence from 43 low- and middle-income countries. Int. J. Epidemiol..

[11] Guo T (2018). The association between ambient PM2.5 exposure and the risk of preterm birth in China: a retrospective cohort study. Sci. Total Environ..

[12] Kirwa K (2021). Preterm birth and PM(2.5) in Puerto Rico: evidence from the PROTECT birth cohort. Environ. Health.

[13] Kirwa K (2019). Low birth weight and PM(2.5) in Puerto Rico. Environ. Epidemiol..

[14] Li X (2017). Association between ambient fine particulate matter and preterm birth or term low birth weight: an updated systematic review and meta-analysis. Environ. Pollut..

[15] Woo B (2019). Residential segregation and racial/ethnic disparities in ambient air pollution. Race Soc. Probl..

[16] Jbaily A (2022). Air pollution exposure disparities across US population and income groups. Nature.

[17] Bureau, U.C. (2023). QuickFacts. https://census.gov/quickfacts/fact/table/PR/PST045222.

[18] Bureau, U.C. (2023). Quickfacts. https://census.gov/quickfacts/.

[19] Frontera-Escudero I (2023). Sociodemographic and health risk factors associated with health-related quality of life among adults living in Puerto Rico in 2019: a cross-sectional study. BMC Public Health.

[20] Cuevas A G (2019). The association between perceived discrimination and allostatic load in the Boston Puerto Rican health study. Psychosom. Med..

[21] Gutierrez C, Dollar N T (2023). Birth and prenatal care outcomes of latina mothers in the trump era: analysis by nativity and country/region of origin. PLoS One.

[22] Brown C C, Moore J E, Tilford J M (2023). Rates of preterm birth and low birthweight: an analysis of racial and ethnic populations. Health Aff. (Millwood).

[23] Parker-Collins W (2023). Pregnancy-related deaths by hispanic origin, United States, 2009–2018. Journal of Women’s Health.

[24] Driscoll A K, Ely D M (2022). Disparities in infant mortality by maternal race and Hispanic origin, 2017-2018. Semin. Perinatol..

[25] Aviles-Santa M L (2020). Funding of hispanic/latino health-related research by the national institutes of health: an analysis of the portfolio of research program grants on six health topic areas. Front. Public Health.

[26] Jirau-Colón H (2021). Distribution of toxic metals and relative toxicity of airborne PM 2.5 in Puerto Rico. Environmental Science and Pollution Research.

[27] Blum J L, Chen L C, Zelikoff J T (2017). Exposure to ambient particulate matter during specific gestational periods produces adverse obstetric consequences in mice. Environ. Health Perspect..

[28] Chatuphonprasert W, Jarukamjorn K, Ellinger I (2018). Physiology and pathophysiology of steroid biosynthesis, transport and metabolism in the human placenta. Front. Pharmacol..

[29] Erickson A C, Arbour L (2014). The shared pathoetiological effects of particulate air pollution and the social environment on fetal-placental development. Journal of Environmental and Public Health.

[30] Luyten L J (2018). Air pollution and the fetal origin of disease: a systematic review of the molecular signatures of air pollution exposure in human placenta. Environ. Res..

[31] Bove H (2019). Ambient black carbon particles reach the fetal side of human placenta. Nat. Commun..

[32] Gao L (2012). Regulation of estradiol and progesterone production by CRH-R1 and -R2 is through divergent signaling pathways in cultured human placental trophoblasts. Endocrinology.

[33] Tuckey R C (2005). Progesterone synthesis by the human placenta. Placenta.

[34] Hahlin M (1990). Single progesterone assay for early recognition of abnormal pregnancy. Hum. Reprod..

[35] Hartwig I R (2013). Sex-specific effect of first-trimester maternal progesterone on birthweight. Hum. Reprod..

[36] Stamatelou F (2009). Abnormal progesterone and corticotropin releasing hormone levels are associated with preterm labour. Ann. Acad. Med. Singap..

[37] Tyson E K, Smith R, Read M (2009). Evidence that corticotropin-releasing hormone modulates myometrial contractility during human pregnancy. Endocrinology.

[38] Smith R (2009). Patterns of plasma corticotropin-releasing hormone, progesterone, estradiol, and estriol change and the onset of human labor. J. Clin. Endocrinol. Metab..

[39] Stamatelou F (2009). Abnormal progesterone and corticotropin releasing hormone levels are associated with preterm labour. Annals of the Academy of Medicine, Singapore.

[40] Yang R (2006). Corticotropin-releasing hormone inhibits progesterone production in cultured human placental trophoblasts. J. Mol. Endocrinol..

[41] Alcantara-Alonso V (2017). Corticotropin-releasing hormone as the homeostatic rheostat of feto-maternal symbiosis and developmental programming in utero and neonatal life. Front. Endocrinol. (Lausanne).

[42] Ellman L M (2008). Timing of fetal exposure to stress hormones: effects on newborn physical and neuromuscular maturation. Dev. Psychobiol..

[43] Kassotaki I (2021). Placental CRH as a signal of pregnancy adversity and impact on fetal neurodevelopment. Front. Endocrinol. (Lausanne).

[44] Leviton A (2016). Brain disorders associated with corticotropin-releasing hormone expression in the placenta among children born before the 28th week of gestation. Acta Paediatr..

[45] Gillman M W (2006). Maternal corticotropin-releasing hormone levels during pregnancy and offspring adiposity. Obesity (Silver Spring).

[46] Wadhwa P D (2004). Placental corticotropin-releasing hormone (CRH), spontaneous preterm birth, and fetal growth restriction: a prospective investigation. Am. J. Obstet. Gynecol..

[47] Linton E A (1993). Corticotropin releasing hormone-binding protein (CRH-BP): plasma levels decrease during the third trimester of normal human pregnancy. J. Clin. Endocrinol. Metab..

[48] Ali E S, Mangold C, Peiris A N (2017). Estriol: emerging clinical benefits. Menopause.

[49] Zhou Y (2022). The steroid hormone estriol (E(3)) regulates epigenetic programming of fetal mouse brain and reproductive tract. BMC Biol..

[50] Kuijper E A (2013). Reproductive hormone concentrations in pregnancy and neonates: a systematic review. Reprod. Biomed. Online.

[51] Dang S (2018). PM(2.5) exposure during pregnancy induces hypermethylation of estrogen receptor promoter region in rat uterus and declines offspring birth weights. Environ. Pollut..

[52] Wang X (2024). Associations between exposure to air pollution and sex hormones during the menopausal transition. Sci. Total Environ..

[53] Meeker J D (2013). Distribution, variability, and predictors of urinary concentrations of phenols and parabens among pregnant women in Puerto Rico. Environ. Sci. Technol..

[54] Cantonwine D E (2014). Urinary phthalate metabolite concentrations among pregnant women in Northern Puerto Rico: distribution, temporal variability, and predictors. Environ. Int..

[55] EPA https://www.epa.gov/outdoor-air-quality-data.

[56] EPA (2015). https://aqs.epa.gov/aqsweb/airdata/FileFormats.html#_daily_summary_files.

[57] Burrough P A, McDonnell R A, Lloyd C D (2015). Principles of Geographical Information Systems.

[58] Aker A M (2019). A repeated measures study of phenol, paraben and Triclocarban urinary biomarkers and circulating maternal hormones during gestation in the Puerto Rico PROTECT cohort. Environmental Health.

[59] Cathey A L (2021). Gestational hormone concentrations are associated with timing of delivery in a fetal sex-dependent manner. Frontiers in Endocrinology.

[60] Hornung R W, Reed L D (1990). Estimation of average concentration in the presence of nondetectable values. Applied Occupational and Environmental Hygiene.

[61] Gelman A (2019). You should (usually) log transform your positive data. https://statmodeling.stat.columbia.edu/2019/08/21/you-should-usually-log-transform-your-positive-data/.

[62] Buuren S, Groothuis-Oudshoorn K (2011). Mice: multivariate imputation by chained equations in R. Journal of Statistical Software.

[63] Bates D (2015). Fitting linear mixed-effects models using lme4. Journal of Statistical Software.

[64] R Core Team (2022). R: A Language and Environment for Statistical Computing.

[65] Li J (2011). Can we improve the spatial predictions of seabed sediments? A case study of spatial interpolation of mud content across the southwest Australian margin. Cont. Shelf Res..

[66] Luechinger S (2014). Air pollution and infant mortality: a natural experiment from power plant desulfurization. J. Health Econ..

[67] Li Z (2019). Impact of ambient PM(2.5) on adverse birth outcome and potential molecular mechanism. Ecotoxicol. Environ. Saf..

[68] Wang C (2017). Suppression of progesterone synthesis in human trophoblast cells by fine particulate matter primarily derived from industry. Environ. Pollut..

[69] Tao S (2023). Maternal exposure to ambient PM(2.5) perturbs the metabolic homeostasis of maternal serum and placenta in mice. Environ. Res..

[70] Naav A (2020). Urban PM2.5 induces cellular toxicity, hormone dysregulation, oxidative damage, inflammation, and mitochondrial interference in the HRT8 trophoblast cell line. Front Endocrinol (Lausanne).

[71] Trivedi S (2012). Fetal-placental inflammation, but not adrenal activation, is associated with extreme preterm delivery. American Journal of Obstetrics & Gynecology.

[72] Morton S (2021). Non-nutritive suck and airborne metal exposures among Puerto Rican infants. Sci. Total Environ..

